# Using Extra Virgin Olive Oil to Cook Vegetables Enhances Polyphenol and Carotenoid Extractability: A Study Applying the *sofrito* Technique

**DOI:** 10.3390/molecules24081555

**Published:** 2019-04-19

**Authors:** José Fernando Rinaldi de Alvarenga, Paola Quifer-Rada, Fernanda Francetto Juliano, Sara Hurtado-Barroso, Montserrat Illan, Xavier Torrado-Prat, Rosa Maria Lamuela-Raventós

**Affiliations:** 1 Department of Nutrition, Food Sciences and Gastronomy, XaRTA, INSA-UB, School of Pharmacy and Food Sciences, University of Barcelona, Carrer Prat de la Riba, 171, 08921 Santa Coloma de Gramenet, Spain; jrinaldi@ub.edu (J.F.R.d.A.); sara.hurtado_17@ub.edu (S.H.-B.); millan@ub.edu (M.I.); xaviertorrado@ub.edu (X.T.-P.); 2 Department of Endocrinology & Nutrition, CIBER of Diabetes and Associated Metabolic Diseases (CIBERDEM), Biomedical Research Institute Sant Pau, Hospital de la Santa Creu i Sant Pau, Carrer de Sant Quintín, 77, 08041 Barcelona, Spain; pquifer@santpau.cat; 3 Department of Agri-food Industry, Food and Nutrition, ‘Luiz de Queiroz’ College of Agriculture, University of São Paulo, Av, Pádua Dias, P.O. Box 9, Piracicaba 13418-900, SP, Brazil; fernanda_ffj@hotmail.com; 4 CIBER of Obesity and Nutrition (CIBEROBN), Institute of Health Carlos III, Av. Monforte de Lemos, 3-5, Pabellón 11, planta 0, 28029 Madrid, Spain

**Keywords:** phenolic compounds, matrix effect, carotenoid isomerization, tomato, onion, garlic, naringenin, lycopene

## Abstract

Olive oil is the main source of fat in the Mediterranean diet and the most frequently used ingredient in Mediterranean cuisine. Cooking with olive oil has been attracting attention because it can act as a food excipient, thereby increasing the bioaccessibility and bioavailability of ingested bioactive compounds. The aim of this study was to understand the effect of cooking with olive oil on the bioactive components in other ingredients (tomato, onion, and garlic) of *sofrito* sauce, a representative model of Mediterranean cuisine. After the cooking process, polyphenols from tomato, onion, and garlic were detected in the olive oil, especially naringenin, ferulic acid, and quercetin, as well as a high content of carotenoid *Z*-isomers, which are more bioavailable than the *E*-isomers. Therefore, traditional Mediterranean cuisine could play an important role in the health-improving effects of the Mediterranean diet.

## 1. Introduction

The Mediterranean diet, characterized by a high intake of phytochemicals from vegetables and legumes, has been correlated with improvements in cardiovascular and metabolic health [1,2]. This association is supported by evidence from the intervention trial PREDIMED, a Mediterranean diet reference study with more than 7000 participants, in which a Mediterranean diet supplemented with extra virgin olive oil (EVOO) reduce the risk of cardiovascular events among high-risk population [2]. Similar results have been observed in other Mediterranean diet clinical trials [3]. However, the health outcomes of a Mediterranean diet are reportedly difficult to reproduce in non-Mediterranean populations, possibly because of different cooking practices [1]. Cooking can negatively affect the phytochemical content of food through oxidation, degradation, or the formation of pro-oxidant compounds. In contrast, it may improve the bioavailability of phytochemicals by altering chemical structures and releasing bioactive compounds from the food matrix [1,4,5].

Traditional Mediterranean cuisine has unique characteristics that could affect the content of bioactive compounds in cooked food [6]. The *sofrito* is a typical technique of lightly frying onion and garlic in EVOO. This sauce is an ingredient used to prepare many Mediterranean dishes and recipes [6,7]. The tomato *sofrito* sauce has been reported to contain 40 different phenolic compounds and a high content of carotenoids [7] and its consumption is associated with improved cardiovascular risk parameters and insulin sensitivity, and activates thermogenesis by browning fat tissue [8,9]. Moreover, tomato *sofrito* consumption is one of a 14-item validated questionnaire used to evaluate the adherence of traditional Mediterranean diet, in which was correlated with low incidence of abdominal fat and obesity and manifestation of less aggressive prostate cancer [10,11,12]. For this reason, we consider *sofrito* a key component of the Mediterranean diet. Olive oil, the predominant source of fat in the Mediterranean diet, displays a singular fatty acid composition, with a higher content of phenolic compounds than other edible oils [13,14]. One of the beneficial effects of olive oil is that it improves the bioavailability of lipophilic compounds such as carotenoids by acting as a food excipient, and enhances nutrient extraction and bioaccessibility [15,16,17,18]. Moreover, cooking with olive oil increases levels of carotenoid *Z*-isomers, which are currently gaining interest due to their higher bioavailability and antioxidant capacity by their geometrical structure compared to carotenoid *E*-isomer form [19]. The polar phenolic compounds, on the other hand, are less soluble in olive oil than carotenoids. Polyphenol bioavailability is low in comparison with other macro- and micronutrients. Only a small percentage of dietary polyphenols (5–10% of total intake) can be directly absorbed in the small intestine, after deconjugation reactions during digestion. The rest (90–95%) may be metabolized by enzymes in the gut microbiome, producing metabolites more easily absorbed in the large intestine, or eliminated through the feces [20]. The absorption of polyphenols could be improved if they are incorporated into olive oil, which would facilitate their entry into epithelial cells by passive diffusion [21]. Recent reports indicate that cooking with olive oil can improve the bioaccessibility and bioavailability of polyphenols [22,23,24], although the underlying mechanisms remain unclear.

The aim of this study is to understand the enhancing effect of olive oil on the extraction of bioactive compounds from other ingredients (tomato, onion, and garlic) in the cooking process, using *sofrito* as a representative model of the Mediterranean diet.

## 2. Results

### 2.1. Identification and Quantification of Phenolic Compounds in the Ingredients

The quantification of phenolic compounds in each ingredient used to prepare the *sofrito* is shown in Table 1. The content of each polyphenol typically found in tomato was quantified by the validated Tomato method, which was also used to quantify polyphenols in onion and garlic. The major polyphenol found in onion was quercetin (34 ± 1 μg/g) [25] followed by p-coumaric (5.9 ± 0.1 μg/g) and ferulic acid (0.62 ± 0.08 μg/g) (Table 1). Polyphenol levels in garlic were much lower, with only two compounds detected, caffeic acid (0.8 ± 0.1 μg/g) and caffeic acid-*O*-hexoside I (0.36 ± 0.02 μg/g) (Table 1).

The content of polyphenols typical from olive oil was quantified in the EVOO as well as in the other ingredients of the tomato-based *sofrito* sauce by Olive oil method. All ingredients were found to contain *p*-coumaric acid and ferulic acid. Additionally, *m*-coumaric and *o*-coumaric acid were detected in tomato and onion, but not in garlic and EVOO. The major polyphenols in EVOO were hydroxytyrosol (14.3 ± 0.8 μg/g), hydroxytyrosol derivatives (oleuropein (5.46 ± 0.08 μg/g), oleuropein derivate I (7.8 ± 0.2 μg/g), hydroxy oleuropein aglycone (3.41 ± 0.04 μg/g) and hydroxydecarboxymethyl oleuropein aglycone (6.6 ± 0.6 μg/g), ligstroside aglycone I (39 ± 1 μg/g), and elenolic acid (7.1 ± 0.5 μg/g) (Table 1). The two methods were used to ensure a suitable selection of candidates for investigating polyphenol migration to the oil fraction in the *sofrito* preparation. To evaluate the migration process, the candidate polyphenols should not be part of the original composition of the EVOO.

### 2.2. Identification and Quantification of Carotenoids in the Ingredients

The analysis of carotenoids in the different *sofrito* fractions revealed the presence of 27 different compounds, only 21 of which were identified, namely lutein, α-carotene, β-carotene, ζ-carotene, lycopene, phytofluene, and apo-β-carotenoids and their isomers (Table 2). The oil fraction had the most diverse carotenoid profile, and all-*E*-lutein, apo-8-β-carotenal, 9,5-*Z*-lycopene, and 9,13-*Z*-lycopene were only quantified in this *sofrito* fraction (Table 3). Tomato was the only ingredient with carotenoids in its composition, showing a high content of all-*E*-lycopene (126 ± 8 µg/g) and some lycopene isomers such as 5-*Z*-lycopene (3.9 ± 0.6 µg/g) and 13-*Z*-lycopene (16 ± 1 µg/g). Also, β-carotene (3 ± 1 µg/g) and α-carotene (1.94 ± 0.09 µg/g) were found.

### 2.3. Identification and Quantification of Phenolic Compounds in the Sofrito Water, Oil and Insoluble Fractions

Using a targeted approach, among all phenolic compounds analyzed only 21 were possible to quantify in the different *sofrito* fractions (Table 3). With the exception of pinoresinol, which remained stable, the level of EVOO polyphenols was considerably reduced in the oil-fraction, possibly because of migration to the food matrix [26] or degradation [27] (Table 3). Carrasco-Pancorbo and co-workers reported an oxidation of phenolic compounds during thermal treatment of EVOO, however pinoresinol was stable during the first hour of processing [27]. In contrast, the oil fraction was enriched with polyphenols from other ingredients, namely caffeic acid, caffeic acid hexoside, chlorogenic acid, naringenin, protocatechuic acid, and quercetin, which were not detected in the EVOO before the cooking process (Table 1). The phenolic compound with the highest transfer efficiency was naringenin (1.7 ± 0.03 μg/g), found at levels close to those in raw tomato (1.9 ± 0.2 μg/g) (Table 1 and Table 3). Ferulic acid was also efficiently transferred to the oil fraction (0.55 ± 0.06 μg/g), where its content was higher than in the initial composition of EVOO (0.0358 μg/g), and its major source were the ingredients garlic (7.2 μg/g) and tomato (1.85 μg/g). Quercetin was not initially detected in EVOO but after cooking showed a concentration of 0.04 ± 0.02 μg/g, onion being the major source of this compound (Table 3).

Analysis of the phenolic content in each fraction expressed in *sofrito* equivalents revealed that the insoluble fraction was the major source of phenolic compounds, with levels very close to the phenolic content of the entire *sofrito*. The second richest fraction in polyphenols was oil, followed by water. The highest content of EVOO phenolics was found in the insoluble fraction, probably the result of frying onions to prepare the *sofrito*. Expressing the results in *sofrito* equivalents, the predominant polyphenols in the oil fraction were naringenin and ferulic acid (0.29 ± 0.06 and 0.10 ± 0.02 μg/g of *sofrito*, respectively). These results would explain the biphasic kinetic curve in plasma described by Martínez-Huélamo and co-workers [24], who reported that the presence of a lipid matrix increased the absorption of ferulic acid and naringenin in volunteers who consumed tomato sauce with olive oil versus tomato sauce without olive oil.

### 2.4. Identification and Quantification of Carotenoids in the Sofrito Water, Oil and Insoluble Fractions

The insoluble fraction had the highest total carotenoid content (53.89 µg/g of *sofrito*) (Table 3), with the all-E-lycopene form (9.6 ± 0.6 µg/g) and the isomers 9-*Z*-lycopene and 5-*Z*-lycopene (9.1 ± 0.7 and 9 ± 1 µg/g, respectively) being predominant. The total carotenoid content in the oil (23.83 µg/g of *sofrito*) (Table 3) was much lower than in the insoluble fraction, but before the cooking process no quantifiable carotenoids were detected in the EVOO. Moreover, oil was the only fraction containing the carotenoids all-*E*-lutein (0.71 ± 0.2 µg/g of *sofrito*), apo-8-β-carotenal (0.40 ± 0.03 µg/g of *sofrito*), 9,13-*Z*-lycopene (0.37 ±0.04 µg/g of *sofrito*), and 9,5-*Z*-lycopene (0.7 ± 02 µg/g of *sofrito*) in quantifiable amounts, which indicates that olive oil can incorporate and solubilize these bioactive compounds. The oil fraction showed almost the same amount of total carotenoids as raw tomato (139.13 versus 150.48 µg/g, respectively), which indicates an efficient migration of carotenoids from the ingredients, given that they were not found in the EVOO before cooking. Another notable result was the presence of carotenoid isomers in the oil fraction that were absent in raw tomato, indicating that they underwent isomerization and incorporation in the oil fraction during the *sofrito* process.

## 3. Discussion

### 3.1. Incorporation of Phenolic Compounds in Extra Virgin Olive Oil (EVOO) during the sofrito Process

The health benefits of dietary phenolic compounds depend on their absorption and bioavailability, which can be affected by differences in cell wall structure, glycoside location in cells, and especially by binding with the food matrix [21]. The cooking process helps to improve bioavailability by promoting chemical structural changes and the release of phytochemicals attached to the food matrix [4]. However, the results of this study show that the major source of polyphenols in the *sofrito* was the insoluble fraction, where they are still bound to the food matrix. Thus, their absorption requires release by the digestion process or the intestinal microbiota. The composition of the microbiota and human intervariabilities can influence this absorption [20]. The enrichment of the insoluble fraction with polyphenols from EVOO could be due to the frying of onions, part of the *sofrito* preparation [26]. Ramírez-Anaya and co-workers [26] studied polyphenols in Mediterranean vegetables prepared with different domestic cooking techniques and identified EVOO polyphenols in sautéed vegetables, indicating a migration from the oil fraction.

In a reverse migration, the *sofrito* oil fraction was found to contain phenolic compounds not present in the EVOO before cooking. Vallverdú-Queralt and co-workers [18], in a study of the effect of oil addition and processing time (15 to 60 min) on the phenolic profile of tomato sauce, found an increase in naringenin when 10% of EVOO was used compared to 5%, with the highest content found at 45 min. Naringenin is trapped in the cuticle of ripe tomato fruit, where it interacts with the insoluble polyesters that constitute tomato peel fiber [28,29]. Thus, mechanical and thermal processing plus the addition of oil could release this compound from the food matrix [23,24,29]. Among the different *sofrito* fractions, approximately 8% of naringenin was found in the oil. The affinity of naringenin for the lipophilic constituents of olive oil may improve its extraction and could contribute to absorption. Once incorporated in the oil fraction, it can enter the epithelial cells by passive diffusion [21,24]. Ban and colleagues [30], in an attempt to improve the bioaccessibility of phenolic compounds, incorporated naringenin in edible oil-producing nanoparticles. Thus protected, the polyphenol showed a good stability and resistance in the harsh conditions of gastrointestinal digestion, demonstrating its affinity for lipophilic environments. Martínez-Huélamo and co-workers [24], comparing the bioavailability of phenolic compounds in raw tomato, tomato sauce, and tomato sauce supplemented with refined olive oil, reported that naringenin content and bioaccessibility were higher after processing than in raw tomato. Additionally, the addition of oil to the tomato sauce enhanced the absorption of naringenin, which supports the possibility that the migration of tomato polyphenols to the EVOO in the *sofrito* preparation may improve their bioavailability [23].

During the cooking process, the hydrolysis of flavonoid glycosides produces free hydroxyl phenol groups, which enhances the lipophilicity of the molecule [13,14]. The molecular structure and the presence of a sugar are important factors for the incorporation of phenolic compounds in the oil matrix. Naringenin, ferulic acid, and quercetin showed good solubility in oil, reflected in the increase in their content in the oil fraction after the *sofrito* preparation.

### 3.2. Incorporation of Carotenoids in Extra Virgin Olive Oil (EVOO) during the sofrito Process

The insoluble fraction of the *sofrito* sauce had the highest carotenoid content, probably due to the presence of chromoplasts in tomato fruit, which remain intact after the cooking process [17] and maintain the chemically stable all-*E*-isoform that predominates in nature [31]. The presence of lycopene and β-carotene isomers in the insoluble fraction confirm that the isomerization reaction starts in the chromoplast cells during the cooking process, which supplies the thermal energy required [32,33,34,35,36]. Carotenoid degradation is avoided if temperatures are maintained between 105 °C and 120 °C [17], which are usual conditions in traditional Mediterranean cuisine. Moreover, thermal treatment helps to release carotenoids by softening cell walls and denaturing the protein-carotenoid complex, allowing the formation of carotenoid isomers and improving their bioaccessibility in the final product [5].

The addition of oil to tomato products helps to partly dissolve lycopene, which is otherwise in an insoluble crystalline form, and may protect lycopene from thermal oxidation during the cooking process and improve its isomerization susceptibility [18,32,35,37]. Moreover, the presence of fat increase the absorption of carotenoids [38,39]. Mutsokoti and co-workers [17] reported that several factors influence the efficiency of carotenoid transfer to the oil phase, including the natural barriers of the food matrix and carotenoid hydrophobicity and structure, the latter being the most important. In the current study, all-*E*-lycopene showed a low solubility in oil, representing 18% of the total lycopene in the oil fraction, whereas *Z*-lycopene forms constituted 82%. Mutsokoti and co-workers [17] reported a less efficient transfer to the oil fraction for lycopene compared to β-carotene. Palmero and colleagues [40] described the same pattern, suggesting that lycopene was entrapped by the matrix, with low transfer to the oil phase and consequently poor micellarization after in vitro digestion. However, neither of the authors discriminated between all-*E*-lycopene and its isomers. The properties and functions of carotenoids are strongly related to the size and shape of the compounds, the flexibility of the *Z*-forms favoring their absorption and transport in comparison with the *E*-form [31].

The presence of multi-*Z*-lycopene isomers in the oil fraction could indicate that this was a stable environment for carotenoid isomers. Multi-*Z*-isomers have been synthesized using a Wittig reaction, obtained by a catalytic reaction with iodine, and isolated from tomatoes, but there is only one report of multi-*Z*-lycopene isomers being obtained by thermal isomerization [41]. In the *sofrito* oil fraction, two di-*Z*-lycopene isomers were characterized, 9,5-*Z*-lycopene and 9,13-*Z*-lycopene. Li and co-workers [42] found di-*Z*-isomers in different tomato cultivars, although the contents were not quantifiable. Lin and Chen [43] found 9,13-*Z*-lycopene and 9,13′-*Z*-lycopene in tomato juice, but at much lower levels (0.17 to 1.03 μg/g) than in the *sofrito* oil fraction. Similarly, Kelebek and co-workers [44], studying the effect of hot and cold breaks in tomato paste processing, reported di-*Z*-lycopene, also at lower levels (0.49 to 0.87 mg/100 g dry matter). The current study differs in that EVOO was employed and phenolic compounds from other ingredients like tomato, onion, and garlic could migrate to the oil fraction. The favorable environment for the production of multi-*Z*-lycopene isomers can be attributed to polyphenols and other chain-braking antioxidants present in different lipid systems. These polyphenols can protect against oxidation in multiphasic systems, like oil/water emulsions, acting together with lipophilic antioxidants such as α-tocopherol [37,45]. Quercetin and naringenin, found in the tomato-based *sofrito*, show good solubility in oil and water, and especially at the oil-water interface, where oxidation takes place [45]. The action of these free radical scavengers could explain the enhanced content of carotenoid *Z*-isomers and di-*Z*-isomers in the oil fraction of the *sofrtio*.

## 4. Materials and Methods

### 4.1. Chemicals and Standards

Acetonitrile, ethanol, methanol, formic acid, and acetic acid were purchased from AppliChem, Panreac Quimica SA (Barcelona, Spain). Hexane, methyl tert-butyl ether (MTBE), all-*E*-α-carotene, all-*E*-β-carotene, all-*E*-lutein, all-*E*-lycopene caffeic acid, *p*-coumaric acid, chlorogenic acid, ferulic acid, isolariciresinol, larisiresinol, luteolin, naringenin, oleuropein, pinoresinol, protocatchuic acid, quercetin, rutin, and secoisolariciresinol were purchased from Sigma-Aldrich (St. Louis, MO, USA). Narigenin-7-*O*-glucoside, hydroxytyrosol, and ethyl gallate were acquired from Extrasynthese (Genay, France), vanillic acid and apigenin from Fluka (St. Louis, MO, USA), and verbascoside from HWI Analytic. C18 ODS SPE bulk sorbent was purchased from Agilent Technologies (Santa Clara, CA, USA). Ultrapure water was obtained using a Milli-Q purification system (Millipore, Bedford, MA, USA).

### 4.2. Material

To prepare the *sofrito* sauce, we used the tomato variety “pera” (*Lycopersicon esculentum* Mill, c. v. Pera) because it is the traditional variety for making tomato sauce and *sofrito*, and moreover it has high content of polyphenols compared to other varieties [46]. They were bought from Grupo Almería (La Cañada, Almería, Spain) from the same batch and had similar diameter (57–67 mm) to avoid composition and climate variability. Olive oil certificated as Extra Virgin category (EVOO) was kindly provided by Manuel Heredia Halcón (Cortijo De Suerte Alta, Albendin-Baena-Córdoba, Córdoba, Spain). Onions and garlic were bought from Ametller Origen to use the products from the same producer to avoid variability, since they provide high quality local products (Barcelona, Spain).

### 4.3. Home-Cooking sofrito Process

In order to study the incorporation of bioactive compounds in EVOO during the cooking process, *sofrito* tomato sauce was chosen as a representative model of traditional Mediterranean cuisine. The *sofrito* recipe was based on previous results of a study applying a full factorial design to evaluate the effect of EVOO, onion, and garlic contents on the carotenoids in a tomato-based *sofrito* sauce (18). The tomato *sofrito* sauce that was cooked in triplicate using 100g of EVOO, 400g of onion, 40g of garlic, and 460g of tomato is the formulation that showed higher content of carotenoids (18).

The tomato-based *sofrito* was prepared at the Food and Nutrition Torribera Campus, University of Barcelona (Santa Coloma de Gramenet, Spain). Tomatoes, garlic, and onion were washed, tomato and onions were cut into small pieces (approximately 5mm), garlic were mixed in a blender (R5 Plus, Robot Coupe^®^, Montceau, Bourgogne, France) and weighed. The bioactive compounds of the ingredients were analyzed after the cutting process to avoid the effect of the process. The *sofrito* cooking process was according to traditional Mediterranean cuisine: in an uncovered pan (24 cm diameter, 15 cm height, 6.3 L volume, 1.59 mm thicknesss, and made of inox 18/10, Paderno, Orfengo, Italy), the EVOO was heated on an electrical cooking plate (180 mm diameter, 1500 W, model Encimera EM/30 2P, Teka^®^, Madrid, Spain) using a potency of 4 (of a range from 1 to 6) for 1 min. After that, the onion and garlic were added and fried for 1 min, and then the tomato was added. From this point the cooking process was timed and the potency was reduced to 2 for low heat cooking following the traditional Mediterranean cuisine and continued for 30 min. The cooking heat was monitored and kept constant throughout the process (100 ± 1 °C) for reproducibility of the samples. After the cooking process, the *sofrito* samples were weighed to quantify the water loss, packaged in plastic vacuum bags, and stored at −25 °C (18).

### 4.4. Isolation of Oil, Water and Insoluble Fractions

In order to isolate the oil, water, and insoluble fractions from the *sofrito* sauce, 20 g of sample was weighed in 50 mL falcon tubes and centrifuged at 20,000 rpm for 30 min at 15 °C, according to the protocol of Palmero et al. (2013) [47]. After the separation of the oil fraction, which consisted of the EVOO added to the formulation, the water fraction, and the insoluble fraction, the different parts were weighed to verify how much each one contributed to the *sofrito* composition.

### 4.5. Extraction and Analysis of Polyphenols

#### 4.5.1. Polyphenol Extraction

The solid fraction and ingredients used before cooking (0.5 g) were extracted with 5 mL of methanol:ultra-pure water (80:20, *v*/*v*) with 0.1% of formic acid for 1 min, sonicated for 10 min in an ice bath, and centrifuged at 4000 rpm for 15 min at 4 °C (Sigma 1-16KL, Sigma, Osterode am Harz, Germany). The supernatant was transferred into a tube and the extraction was repeated. After that, the supernatants were combined and evaporated using a vacuum evaporator (miVac DNA concentrator, Genevac LTD, Warminster, England). The residue was suspended in up to 2 mL of ultra-pure water with 0.1% of formic acid [7].

The oil fraction (1 g) was diluted with 1 mL of *n*-hexane and after the addition of 2 mL of methanol, it was homogenized and centrifuged at 3000 rpm for 3 min at 4 °C. The nonpolar and polar phases were separated and extracted again with 1 mL of *n*-hexane and 2 mL of methanol, respectively. The polar phases were combined, cleaned up with 50 mg of C18 to remove the polar fatty acids, homogenized for 1 min and centrifuged. In order to check for the presence of residual lipids, the extract was placed in an ultra-freezer for 15 min and a visual inspection was performed. The clean liquid was evaporated until dryness using a vacuum evaporator and the residue was suspended in up to 2 mL of ultra-pure water with 0.1% of formic acid [48].

The water fraction (1 g) was weighed and centrifuged at 4000 rpm for 15 min at 4 °C. The supernatant was transferred to a 2 mL volumetric flask to which ultra-pure water with 0.1% of formic acid was added. All the extracts were filtered thought a 0.22 µm polytetrafluoroethylene (PTFE) filter into a 2 mL amber vial for HPLC and stored at −80 °C until analysis.

#### 4.5.2. Polyphenol Analysis by UPLC-ESI-QqQ-MS/MS

The polyphenols typically found in tomato, onion and garlic were identified and quantified by a UHPLC-MS/MS method validated for tomato polyphenols (Tomato method) [49] and EVOO polyphenols were analyzed using a another method more approprieate for them (Olive oil method) [50]. A UPLC Acquity system equipped with a binary pump, autosampler, and oven from Waters (Milford, MA, United States) with a BEH C18 column (50 mm × 2.1 mm) i.d., 1.7 µm (Waters, Milford, MA, United States) was used. The injection volume was 10 µL, the samples were maintained at 4 °C and the column at 30 °C.

The separation of tomato polyphenols (Tomato method) was carried out with a phase A consisting of acetonitrile (0.1% formic acid) and phase B of water (0.1% formic acid). A gradient elution was applied as follows: 0 min, 10%A; 0.5 min, 10%A, 1.5 min, 15% A; 2.0 min, 20% A; 2.5 min, 50%A; 3.0 min, 100% A; 3.5 min 100% A, and 4.5 min, 10% A. For the EVOO polyphenols (Olive oil method), the separation was performed with a phase A consisting of acetonitrile (0.2% acetic acid) and phase B of water (0.2% acetic acid). The gradient elution was: 0 min, 5%A; 2.5 min, 5%A; 12.5 min, 40%A; 12.6 min, 100%A; 13.5 min, 100%A; 13.6 min, 5%A, and 15.0 min, 5%A. A flow rate of 400 µL/min was applied for both methods.

An API 3000 triple quadrupole mass spectrometer (ABSciex, Framingham, MA, USA), coupled with a Turbo Ionspray source in negative ion mode was used for the MS/MS analysis. The settings of the Turbo Ionspray were: capillary voltage, -3500 V; nebulizer gas (N2), 10 (arbitrary units); curtain gas (N_2_), 12 (arbitrary units); collision gas (N_2_), 4 (arbitrary units); and drying gas (N_2_) heated at 400 °C introduced at a flow rate of 8000 cm^3^/min. To improve the detection, the declustering potential, focusing potential, and collision energy were optimized for each compound by direct infusion experiments: 10 ppm individual standard solutions, dissolved in 1:1 (*v*/*v*) mobile phase, were infused at a flow rate of 5 µL/min with a model syringe pump (Harvard Apparatus, Holliston, MA, USA). A full-scan data acquisition in profile mode, scanning from 100 to 800 *m*/*z*, was used in cycles of 2 s with a step size of 0.1 u and a pause between each scan of 0.002 s (Table 1).

The polyphenols were quantified using multiple reaction monitoring mode (MRM), tracking the transition of parent ion and product ions specific for each compound. The quantification was performed using the internal standard method, applying ethyl gallate as the internal standard, and quantified with calibration curves related to the corresponding standard. The limit of quantification (LoQ) was: 1.13 ng/mL for chlorogenic acid; 9.75 ng/mL for caffeic acid, protocatechuic acid, ferulic acid and verbascoside; 27.0 ng/mL for naringenin-7-O-glucoside, *p*-coumaric, isolariciresinol, lariciresinol; 75.0 ng/mL naringenin, quercetin, rutin, apigenin, luteolin, hydroxytyrosol, and oleuropein. When commercial standards were not available, the compound was quantified using a standard of the same class of polyphenol. The results were expressed as µg/g of fraction.

### 4.6. Extraction and Analysis of Carotenoids

#### 4.6.1. Carotenoid Extraction

The solid fraction and ingredients (0.5 g) and water fractions (1 g) were weighed and homogenized with 5 mL of ethanol *n*-hexane (4:3, *v*/*v*), and then sonicated for 10 min in an ice bath and centrifuged at 4000 rpm for 20 min at 4 °C. The apolar phase was separated in a different flask and the extraction was repeated until it was colorless. All the supernatants were combined and evaporated to dryness using a vacuum concentrator (miVac DNA concentrator, Genevac LTD, Warminster, England). The residue was suspended in 1 mL of MTBE, filtered using a 0.22 µm PTFE filter, and stored in a 2 mL amber vial at −80 °C [36].

The oil fraction (1 g) was extracted using 3 mL cold acetone (8 °C), then sonicated for 10 min in an ice bath and centrifuged in the same conditions. The extract was kept at freezer temperature (−25 °C) for 30 min and the separation of acetone from residual fat was visually checked. After that, the extraction was repeated until it was colorless. The carotenoids were transferred to 2 mL of hexane and washed 4 times with 5 mL of ultrapure water to remove the acetone. The hexane was evaporated until dryness using a vacuum concentrator and the residue was suspended in 1 mL of MTBE, filtered with a 0.22 µm PTFE filter in a 2 mL amber vial, and stored at −80 °C.

#### 4.6.2. Carotenoid Analysis by HPLC-DAD and HPLC-APCI-QqQ-MS/MS

The carotenoid analysis was based on the procedure of Vallverdú-Queralt et al. (2015) [36] with modifications. An HPLC system (HP1100 HPLC system, Hewlett-Packard, Waldbronn, Germany) equipped with a quaternary pump and autosampler and coupled with a diode array detector (DAD G1315B) was used. Separation was carried out using a C30 250 × 4.6 mm, 5 μm column (YMCTM, Water Co., Milford, MA, USA), with a flow rate of 600 μL/min at 25 °C. The injection volume was 20 μL. The mobile phase consisted of methanol (A), MTBE (B) and water (C). A gradient was used to separate the carotenoid compounds under the following conditions: 0 min, 70% A; 15 min, 20% A; 30 min, 6% A; 31 min, 6% A; 33 min, 70% A; and 43 min, 70% A. Water was kept constant at 4% throughout the analysis. The DAD detector was applied in the range of 350 to 600 nm and the chromatograms were acquired at a 450 nm wavelength.

A QTRAP4000 triple quadrupole mass spectrometer (Sciex, Foster City, CA, USA) equipped with an APCI ionization source operating in positive-ion and multiple reaction monitoring mode was used to confirm the identification. The carotenoids were separated using the same column and a different mobile phase was applied: methanol (A) and MTBE:methanol (80:20, *v*/*v*) (B), both fortified with 0.7 g/L of ammonium acetate and 0.1% acetic acid. The linear gradient of A was: 0 min, 90%; 10 min, 75%; 20 min, 50%; 25 min, 30%; 35 min, 10%; 37 min, 6%; 39 min, 90%; and 50 min, 90%, with a flow rate of 600 μL/min. The mass spectrometer conditions were: entrance potential (EP), 10 V; collision cell exit potential (CXP), 15 V; temperature source, 400 °C, curtain gas, 20 psi, ion source gas 1 (GS1), 45 psi; ion source gas 2 (GS2), 0 psi. Declustering potential (DP) was selected for the carotenoids according to previously established conditions [51].

The identification was based on retention time, chromatography with standards, mass spectra, and UV/VIS absorption spectra: λ_max_, spectral fine structure (%III/II) and peak cis intensity (%A_b_/A_II_). The carotenoids were quantified by HPLC-DAD with external calibration curves of α-carotene, β-carotene and lycopene with seven concentration levels. The limit of quantification (LoQ) was 0.2 for lycopene and 0.4 ppm for α-carotene and β-carotene. The Z-isomers were quantified with the calibration curve of the corresponding E-form. The results were expressed as µg/g of fraction.

### 4.7. Statistical Analysis

The Shapiro–Wilk test was used to check data normality. The statistical differences between EVOO before and after cooking were analyzed by R v 3.4.4 using the *t*-test, and the differences between different fractions and tomato *sofrito* sauce by Dunn’s Kruskal–Wallis multiple comparisons.

## 5. Conclusions

The use of olive oil in Mediterranean cuisine may improve the extractability of bioactive compounds such as polyphenols and carotenoids from food matrix. The migration of polyphenols, such as naringenin, ferulic acid, and quercetin, which are compounds not detected in olive oil, to the oil fraction during the *sofrito* preparation may enhance their bioacessibility and bioavailability. Carotenoids from tomato can also be transferred to the oil fraction, especially *Z*-isomers, due to their structure and solubility, and this could improve their bioacessibility. The presence of these compounds in oil fraction after the cooking process indicates their stability in oil matrix avoiding oxidation. The beneficial effects of the Mediterranean diet may be due not only to the consumption of certain foods, but also cooking techniques such as *sofrito*, which can help the extraction of carotenoids and phenolic compounds from food matrix, and could contribute to its bioacessibility, bioavailability, and health effects.

## Figures and Tables

**Table 1:** Polyphenols monitored and quantified in the ingredients of the tomato-based *sofrito* sauce expressed in µg/g of ingredient.

Compound	rt	MRM (*m*/*z*)	DP (V)	FP (V)	EP (V)	CE (V)	T	O	G	E
**Tomato Method ^A^**										
caffeic acid-*O*-hexoside I	0.33	341→179	−40	−170	−10	−20	4.7 ± 0.2	0.30 ± 0.03	0.36 ± 0.02	n.d.
caffeic acid-*O*-hexoside II	0.51	341→179	−40	−170	−10	−20	n.d.	n.d.	n.d.	n.d.
chlorogenic acid *	0.53	353→191	−50	−180	−10	−20	4.4 ± 0.4	n.d.	n.d.	n.d.
protocatechuic *	0.54	153→109	−40	−150	−10	−20	0.0014 ± 0.0001	n.d.	n.d.	n.d.
coumaric acic-*O*-hexoside	0.55	325→163	−40	−150	−10	−25	1.58 ± 0.09	n.d.	n.d.	n.d.
ferulic acid-*O*-hexoside	0.59	355→193	−40	−170	−10	−25	4.5 ± 0.4	n.d.	n.d.	n.d.
caffeic acid *	0.69	179→135	−40	−170	−10	−20	1.36 ± 0.03	n.d.	0.8 ± 0.1	n.d.
Rutin *	0.74	609→300	−60	−230	−10	−50	2.94 ± 0.05	n.d.	n.d.	n.d.
ethyl gallate * (IS)	1.01	197→169	−60	−200	−10	−25	-	-	-	-
naringenin-7-*O*-glucoside *	1.39	433→271	−50	−280	−10	−30	0.076 ± 0.009	n.d.	n.d.	n.d.
dicaffeoylquinic acid	1.41	515→353	−50	−180	−10	−20	0.61 ± 0.02	n.d.	n.d.	n.d.
Quercetin *	2.08	301→151	−60	−210	−10	−30	0.236 ± 0.002	34 ± 1	n.d.	n.d.
naringenin *	2.65	271→151	−50	−190	−10	−30	1.9 ± 0.2	n.d.	n.d.	n.d.
**Olive Oil Method ^B^**										
1-acetoxypinoresinol	-	451→325	−60	−180	−8	−25	n.d.	n.d.	n.d.	n.d.
dihydroxyoleuropein aglycone (DHOA)	-	409→180	−30	−140	−10	−30	n.d.	n.d.	n.d.	n.d.
dihydroxyphenylacetic acid	-	167→123	−40	−170	−10	−10	n.d.	n.d.	n.d.	n.d.
hydroxytyrsol acetate. (3,4-DHPEA-AC II)	-	195→153	−30	−140	−10	−30	n.d.	n.d.	n.d.	n.d.
*p*-coumaroyl-6-oleoside	-	535→427	v30	−140	−10	−30	n.d.	n.d.	n.d.	n.d.
tyrosol	-	137→106	−25	−90	−10	−20	n.d.	n.d.	n.d.	n.d.
hydroxytyrosol-*O*-glucoside	1.01	315→153	−40	−250	−10	−20	n.d.	n.d.	n.d.	0.038 ± 0.004
Hydroxytyrosol *	1.13	153→123	−40	−250	−10	−20	n.d.	n.d.	n.d.	14.3 ± 0.8
*p*-coumaric acid *	1.57	163→119	−40	−150	−10	−25	0.108 ± 0.007	5.9 ± 0.1	0.057 ± 0.008	0.200 ± 0.006
hydroxycarboxymethyl elenolic acid I (HCM-EA I)	1.65	199→155	−30	−140	−10	−30	n.d.	n.d.	n.d.	n.d.
vanillic acid *	2.56	167→123	−30	−200	−10	−30	n.d.	n.d.	n.d.	n.d.
*m*-coumaric acid *	3.26	163→119	−40	−150	−10	−25	0.71 ± 0.02	0.014 ± 0.001	n.d.	n.d.
ethyl gallate * (IS)	4.62	197→169	−60	−200	−10	−25	-	-	-	-
*o*-coumaric acid *	4.75	163→119	−40	−150	−10	−25	1.9 ± 0.1	0.012 ± 0.002	n.d	n.d
ferulic acid *	5.15	193→134	−40	−170	−10	−20	1.85 ± 0.08	0.62 ± 0.08	7.2 ± 0.3	0.0358 ± 0.0004
hydroxytyrsol acetate. (3,4-DHPEA-AC)	5.69	195→180	−30	−140	−10	−30	n.d.	n.d.	n.d.	n.d.
hydroxyelenolic acid	6.02	257→137	−30	−140	−10	−30	n.d.	n.d.	n.d.	1.17 ± 0.2
elenolic acid *	6.31	241→127	−30	−140	−10	−30	n.d.	n.d.	n.d.	7.1 ± 0.5
verbascoside *	6.4	623→161	−90	−210	−10	−50	n.d.	n.d.	n.d.	n.d.
isolariciresinol *	6.44	359→344	−60	−100	−13	−30	n.d.	n.d.	n.d.	n.d.
lactone	7.16	321→185	−40	−250	−10	−20	n.d.	n.d.	n.d.	0.68 ± 0.06
secoisolariciresinol	7.24	361→165	−60	−50	−6	−35	n.d.	n.d.	n.d.	
hydroxycarboxymethyl elenolic acid II (HCM-EA II)	7.3	199→155	−30	−140	−10	−30	n.d.	n.d.	n.d.	n.d.
hydroxydecarboxymethyl oleuropein aglycone (HDCM-OA)	7.32	335→199	−30	−140	−10	−30	n.d.	n.d.	n.d.	6.6 ± 0.6
lariciresinol *	7.45	359→329	−40	−100	v4	−15	n.d.	n.d.	n.d.	n.d.
hydroxy oleuropein aglycone I (HOA I)	7.6	393→257	−30	−140	−10	−30	n.d.	n.d.	n.d.	3.41 ± 0.04
oleuropein *	7.68	539→275	−30	−140	−10	−30	n.d.	n.d.	n.d.	5.46 ± 0.08
oleuropein aglycone (3,4-DHPEA-EA) II	7.91	377→307	−30	−140	−10	−30	n.d.	n.d.	n.d.	n.d.
luteolin *	8.15	285→133	−100	−340	−10	−50	n.d.	n.d.	n.d.	0.89 ± 0.06
decarboxylmethyl oleuropein aglycone (3,4-DHPEA-EDA)	8.4	319→181	−30	−140	−10	−30	n.d.	n.d.	n.d.	0.114 ± 0.005
pinoresinol *	8.58	357→151	−60	−180	−8	−25	n.d.	n.d.	n.d.	0.089 ± 0.001
oleuropein derivative II	8.64	377→307	−30	−140	−10	−30	n.d.	n.d.	n.d.	7.8 ± 0.2
hydroxy oleuropein aglycone II (HOA II)	8.8	393→257	−30	−140	−10	−30	n.d.	n.d.	n.d.	n.d.
oleuropein derivative III	8.92	377→307	−30	−140	−10	−30	n.d.	n.d.	n.d.	n.d.
oleocanthal (4-HPEA-EDA)	9.0	303→165	−25	−90	−7	−15	n.d.	n.d.	n.d.	n.d.
ligstroside aglycon I	9.01	361→291	−30	−140	−10	−30	n.d.	n.d.	n.d.	39 ± 1
apigenin *	9.11	269→117	−70	−200	−10	−50	n.d.	n.d.	n.d.	0.36 ± 0.03
oleuropein derivative I	9.14	377→241	−30	−140	−10	−30	n.d.	n.d.	n.d.	5.46 ± 0.08
ligstroside aglycon II	10.0	361→291	−30	−140	−10	−30	n.d.	n.d.	n.d.	30.9 ± 0.6
oleuropein aglycone (3,4-DHPEA-EA)	10.0	377→307	−30	−140	−10	−30	n.d.	n.d.	n.d.	n.d.
oleuropein derivative IV	10.3	377→307	−30	−140	−10	−30	n.d.	n.d.	n.d.	n.d.
ligstroside aglycon III	11.2	361→291	−30	−140	−10	−30	n.d.	n.d.	n.d.	17.3 ± 0.6
methyl oleuropein aglycone (methyl 3,4-DHPEA-EA)	11.2	391→255	−30	−140	−10	−30	n.d.	n.d.	n.d.	0.045 ± 0.001

^A^ Tomato method was used to quantify phenolic compounds typically from tomato, onion and garlic. ^B^ Olive oil method was used to quantify phenolic compounds typically from olive oil. * Compounds confirmed by standards. Results were expressed in µg/g of ingredient by mean ± SD, n = 3. rt (retention time); MRM (multiple reaction monitoring); DP (declustering potential); FP (focusing potential); EP (entrance potential); CE (collision energy); T (tomato); O (onion); G(garlic); E (extra virgin olive oil); n.d. (not detected).

**Table 2:** Carotenoid identification in the *sofrito* oil fraction.

Peak	Compound	rt (min)	λ_max_ (nm)	%III/II	%A_b_/II	[M + H]^+^ (*m*/*z*)	MS^2^ ion products (*m*/*z*)
1	all-*E*-lutein *	10.51	(420), 445, 476	40	14	551 [M + H-18]	429 [M − 122]
2	n.i.	11.12	445, 472			-	-
3	n.i.	11.93	(330), (347), (416), 440, 470	41	21	-	-
4	n.i.	12.69	(330), (345), (422), 440, 468	30	15	-	-
5	n.i.	14.31	434, 456			-	-
6	n.i.	15.20	430, 452, 485	38	4	-	-
7	Apo-10-β-carotenal	15.58	448			-	n.d.
8	Apo-8-β-carotenal *	15.99	460			417	325 [M − 92]
9	all-*E*-α-carotene *	16.39	425, 445,475			537	444 [M − 92]
10	*Z*-phytofluene	16.43	332, 349, 368	72		543	406 [M + H − 137]
11	phytofluene	16.79	332, 349, 368	90		543	406 [M + H − 137]
12	all-*E*-ζ-carotene	17.31	385, 401, 420	104		541	472 [M + H − 69]
13	*Z*-β-carotene	17.05	418, 437, 461	13			444 [M − 92]; 413 [ M − 123]
14	13-*Z*-β-carotene	17.91	(335), (347), 448, 470	28	32	537	444 [M − 92]; 413 [ M − 123]
15	all-*E*-β-carotene *	18.95	(425), 453, 478	28		537	444 [M − 92]; 413 [ M − 123]
16	9-*Z*-β-carotene *	19.71	(350), 426, 453, 480	28	28	537	444 [M − 92]; 413 [ M − 123]
17	n.i.	20.37	(350), 426, 453, 480		20	537	444 [M − 92]; 413 [ M − 123]
18	n.i.	21.34	(350), 455, 485				n.d.
19	9,13-*Z*-lycopene	22.17	(348), (362), 437, 460, 488	47	28	537	444 [M − 92]; 413 [ M − 123]
20	15-*Z*-lycopene	23.11	(348), (362), 438, 461, 490	48	69	537	444 [M − 92]; 413 [ M − 123]
21	all-*E*-γ-carotene	23.41	(364), 438, 461, 491	58		537	444 [M − 92]
22	13-*Z*-lycopene	23.89	(348), (362), 440, 466, 495	49	40	537	444 [M − 92]; 413 [ M − 123]
23	9,5-di-*Z*-lycopene	24.57	(348), (362), 438, 460, 489	54	22	537	444 [M − 92]; 413 [ M − 123]
24	9-*Z*-lycopene	26.88	(348), (362), 441, 467, 497	70	17	537	444 [M − 92]; 413 [ M − 123]
25	7-*Z*-lycopene	27.23	(348), (363), 441, 467, 497	70	17	537	444 [M − 92]; 413 [ M − 123]
26	all-*E*-lycopene *	31.29	447, 472, 503	74	0	537	444 [M − 92]; 413 [ M − 123]
27	5-*Z*-lycopene	31.89	(365), 446, 472, 503	74	5	537	444 [M − 92]; 413 [ M − 123]

λ_max_ (UV/VIS absorption spectra); %III/II (spectral fine structure); %A_b_/AII (peak cis intensity); n.i. (not identified); n.d. (not detected); * Compounds confirmed by standards.

**Table 3:** Quantification of polyphenols in water, oil and insoluble fractions of *sofrito* expressed in μg/g of *sofrito* or ingredients.

Compound	μg/g of Ingredient or Fraction ^A^		μg/g of *sofrito* ^B^			
	EVOO	Oil Fraction	*Sofrito*	Water	Oil	Insoluble
**Polyphenols**						
apigenin	0.36 ± 0.03	0.16 ± 0.02 ***	1.64 ^a^ ±0.007	0.008 ^c^ ± 0.002	0.028 ^b^ ± 0.006	1.5 ^a^ ± 0.1
elenolic acid	22 ± 2	0.083 ± 0.06 ***	0.110 ^b^ ± 0.004	0.010 ^c^ ± 0.002	0.014 ^c^ ± 0.002	1.49 ^a^ ± 0.08
ferulic acid	0.0358 ± 0.00004	0.55 ± 0.06 ***	0.64 ^b^ ± 0.6	0.007 ^c^ ± 0.002	0.10 ^c^ ± 0.02	0.75 ^a^ ± 0.09
ligstroside I	39 ± 1	1.8 ± 0.3 ***	0.109 ^c^ ± 0.004	0.007 ^c^ ± 0.001	0.31 ^b^ ± 0.07	0.6 ^a^ ± 0.1
luteolin	0.89 ± 0.06	0.13 ± 0.01 **	0.169 ^b^ ± 0.007	0.013 ^c^ ± 0.004	0.022 ^c^ ± 0.002	2.9 ^a^ ± 0.4
*m*-coumaric	n.d.	<LoQ	<LoQ	0.018 *** ± 0.004	<LoQ	0.40 *** ± 0.02
*o*-coumaric	n.d.	0.08 ± 0.01	<LoQ	0.020 ^b^ ± 0.004	0.015 ^b^ ± 0.003	0.54 ^a^ ± 0.06
*p*-coumaric	0.200 ± 0.006	0.08 ± 0.01 **	0.12 ^a^ ± 0.02	0.0019 ^b^ ± 0.0004	0.015 ^b^ ± 0.003	0.16 ^a^ ± 0.02
oleuropein	5.46 ± 0.08	1.14 ± 0.08 ***	0.111 ^b^ ± 0.004	0.026 ^b^ ± 0.008	0.20 ^b^ ± 0.03	2.5 ^a^ ± 0.2
pinoresinol	0.089 ± 0.001	0.09 ± 0.01	0.35 *** ± 0.20	<LoQ	0.016 *** ± 0.003	<LoQ
caffeic acid	n.d.	0.032 ± 0.001	1.8 ^a^ ± 0.2	0.006 ^c^ ± 0.002	0.0055 ^c^ ± 0.0003	0.21 ^b^ ± 0.01
caffeic acid-*O*-hexoside	n.d.	0.0183 ± 0.0007	2.6 ^a^ ± 0.3	0.0023 ^c^ ± 0.0005	0.0032 ^c^ ± 0.0002	0.16 ^b^ ± 0.02
chlorogenic acid	n.d.	0.0012 ± 0.0003	5.4 ^a^ ± 0.6	0.0025 ^b^ ± 0.0009	<LoQ	0.064 ^b^ ± 0.008
dicalfeoylquinic acid	n.d.	n.d.	0.35 ^a^ ± 0.01	0.0018 ^c^ ± 0.0004	n.d.	0.077 ^b^ ± 0.002
ferulic acid-*O*-hexoside	n.d.	n.d.	6 ^a^ ± 2	0.05 ^c^ ± 0.02	n.d.	1.2 ^b^ ± 0.2
naringenin	n.d.	1.7 ± 0.03	2.8 ^b^ ± 0.1	0.021 ^c^ ± 0.006	0.29 ^c^ ± 0.06	3.4 ^a^ ± 0.6
naringenin-7-*O*-glucoside	n.d.	n.d.	0.025 ^b^ ± 0.003	0.010 ^b^ ± 0.003	n.d.	0.60 ^a^ ± 0.06
protocatechuic acid	n.d.	0.009 ± 0.001	n.d.	0.003 ^b^ ± 0.001	0.0016 ^b^ ± 0.0002	0.06 ^a^ ± 0.02
quercetin	n.d.	0.04 ± 0.02	5 ^b^ ± 1	0.03 ^c^ ± 0.01	0.007 ^c^ ± 0.003	10 ^a^ ± 3
rutin	n.d.	n.d.	2.6 ^a^ ± 0.1	0.023 ^c^ ± 0.006	n.d.	0.62 ^b^ ± 0.08
**Carotenoids**						
all-*E*-lutein	<LoQ	4.2 ± 0.2	n.d.	n.d.	0.71 ± 0.2	n.d.
Apo-8-β-carotenal	n.d.	2.34 ± 0.01	n.d.	n.d.	0.40 ± 0.03	n.d.
all-*E*-α-carotene	n.d.	2.14 ± 0.03	3.6 ^a^ ± 0.1	0.020 ^d^ ± 0.005	0.37 ^c^ ± 0.03	1.61 ^b^ ± 0.06
13-*Z*-β-carotene	n.d.	3.1 ± 0.2	<LoQ	0.022 ^c^ ± 0.005	0.53 ^b^ ± 0.08	1.98 ^a^ ± 0.08
all-*E*-β-carotene	<LoQ	8.0 ± 0.8	7.3 ^a^ ± 0.5	0.029 ^c^ ± 0.007	1.4 ^b^ ± 0.2	6.2 ^a^ ± 0.6
9-*Z*-β-carotene	n.d.	6.5 ± 0.4	3.4 ^b^ ± 0.2	0.023 ^d^ ± 0.06	1.1 ^c^ ± 0.2	6.4 ^a^ ± 0.6
9,13-*Z*-lycopene	n.d.	2.15 ± 0.06	n.d.	n.d.	0.37 ± 0.04	n.d.
15-*Z*-lycopene	n.d.	2.6 ± 0.3	<LoQ	n.d.	0.45 *** ± 0.08	2.9 *** ± 0.2
13-*Z*-lycopene	n.d.	8 ± 1	14.6 ^a^ ± 0.7	0.035 ^d^ ± 0.008	1.4 ^c^ ± 0.4	7.1 ^b^ ± 0.6
9,5-*Z*-lycopene	n.d.	4.1 ± 0.5	n.d.	n.d.	0.7 ± 0.2	n.d.
9-*Z*-lycopene	n.d.	40 ± 4	25 ^a^ ± 2	0.040 ^c^ ± 0.009	7 ^b^ ± 1	9.1 ^b^ ± 0.7
all-*E*-lycopene	n.d.	20 ± 2	46.8 ^a^ ± 0.7	0.038 ^d^ ± 0.009	3.4 ^c^ ± 0.7	9.6 ^b^ ± 0.6
5-*Z*-lycopene	n.d.	36 ± 3	42 ^a^ ± 2	0.004 ^d^ ± 0.01	6 ^c^ ± 1	9 ^b^ ± 1
Total carotenoids	-	139.13	142.7	0.221	23.83	53.89

EVOO (extra virgin olive oil); <LoQ (below the limit of quantification); n.d. (not detected). Results were expressed in mean ± SD; n = 3. ^A^ Statistical analysis between EVOO and the oil fraction (expressed in g of oil) applying t-test. Values in a row with symbols show significant differences from * *p* ≤ 0.05, ** *p* ≤ 0.01 and *** *p* ≤ 0.001, ^B^ Statistical analysis between Sofrito and fractions (expressed in g of sofrito) applying Dunn’s Kruskal–Wallis multiple comparison. Values in a row with different letter show significant differences (*p* ≤ 0.05).
